# Comparing Peer-Taught Student Tutors to Faculty-Taught Student Tutors in Educating Medical Students on Musculoskeletal Ultrasound

**DOI:** 10.7759/cureus.59166

**Published:** 2024-04-27

**Authors:** Matthew Aquino, Jedd Santamaria, Ebraheem Quadri, Benjamin Riegsecker, Jeffrey Li, Jane Kim, Fauzia Nausheen, Vy Han

**Affiliations:** 1 Emergency Medicine, California University of Science and Medicine, Colton, USA; 2 Anesthesiology, California University of Science and Medicine, Colton, USA; 3 Radiology, California University of Science and Medicine, Colton, USA; 4 Internal Medicine, California University of Science and Medicine, Colton, USA; 5 Medical Education, California University of Science and Medicine, Colton, USA

**Keywords:** peer-assisted learning, medical school education, preclinical education, ultrasound anatomy, musculoskeletal imaging, ultrasound education

## Abstract

Introduction: In recent years, medical education has witnessed a shift in the integration of ultrasound into the preclinical years of medical school. Given the exponential increase in accessibility to ultrasound technology, students now have the opportunity to create peer learning groups in which ultrasound concepts can be taught from peer to peer, empowering students to work together to integrate ultrasound concepts early in their preclinical education. This project investigates the efficacy of peer-taught student tutors (PTSTs) in imparting the fundamentals of basic ultrasound techniques to first-year medical students in the setting of identifying and labeling upper extremity musculoskeletal (MSK) anatomy.

Methods: First-year medical students were instructed to identify volar forearm structures with an ultrasound probe. Students and instructors were given access to an ultrasound probe, ultrasound gel, an iPad, and a standardized patient. Students were taught either by an ultrasound instructor (UI) or PTST. After a hands-on demonstration by a UI or PTST, participating students were told to take screenshots and label their images as accurately as possible, identifying the aforementioned volar structures on a standardized patient without any feedback. The labeled screenshot images of volar structures were graded based on the ability to clearly visualize the intended structures.

Results: The results of this study compare the efficacy of PTSTs as educators of basic sonographic identification techniques with that of UI faculty members. A chi-square analysis was performed between the images obtained by the UI and PTST students, and there was no statistically significant difference in identification accuracy between the groups (p = 0.7538, 0.1977, 0.1812, 0.301). When using the Mann-Whitney U rank test, there remained no statistically significant difference between the accuracy of the students taught by STs compared to students taught by UIs (p = 0.7744, 0.09538, 0.07547, 0.1846). Another finding showed that students belonging to both teaching groups were generally not able to infer the pathology of volar wrist structures when given pathology identification questions regarding upper extremity ultrasound. Using chi-square with Yates correction, there is no sufficient evidence to justify an association between the ability to answer pathology-based ultrasound questions and instructor type (p = p = 0.6299, 0.8725).

Conclusions: This study supports the interpretation that the capability of first-year medical students to learn novice MSK sonographic identification is independent of whether the educator is a PTST or UI. This interpretation reveals a promising avenue toward the integration of the fundamentals of ultrasound identification early in medical education with little to no concern for the exhaustion of institutional resources. Along with the other well-documented benefits of the utilization of STs in medical school, a peer tutoring system centered on ultrasound skills designed in the way this study describes can be an effective, resource-sparing system that enhances medical students’ sonographic capabilities early in their preclinical years.

## Introduction

In recent years, medical education has witnessed a transformative shift in the integration of ultrasound training into the preclinical years of medical school. This evolution has been driven not only by an exponential rise in accessibility to ultrasound technology but also by the growing recognition of ultrasound as a powerful diagnostic tool that enhances students' understanding of anatomy, pathology, and clinical correlations [1,2]. Many institutions have already implemented the use of ultrasonography to supplement didactic anatomy lectures with significant increases in students’ perceived comfort and confidence in ultrasound skills and bolstering the understanding of anatomical structure [2-6]. As ultrasound technology becomes more accessible and relevant in various medical specialties, the need for proficient ultrasound training among medical students during their preclinical years has become increasingly important and valuable [7].

Limited ultrasound exposure and resource constraints are common challenges faced by medical schools worldwide. However, multiple studies suggest that preclinical medical students can attain a sufficient degree of proficiency in limited ultrasonographic techniques with guidance from proficient teachers [5,6]. In this context, the question arises: can student tutors (STs), trained by their peers, serve as adequate instructors in imparting the fundamental skills of ultrasound to their fellow preclinical students? This research paper seeks to explore and answer this question, shedding light on the effectiveness and feasibility of this peer-driven educational approach. A number of existing studies have sought to measure the effectiveness of student tutoring in medical school with promising results [8-14] showing an overwhelmingly positive reception of students to the effectiveness of learning from their peers. However, no current research exists comparing the effectiveness of peer-taught student tutors (PTSTs) as educators of ultrasound techniques.

While ultrasound's potential to revolutionize medical education is widely acknowledged, the allocation of resources and teaching hours for ultrasound training remains a challenge, particularly during the early preclinical years of medical school. Financial burden and the rigorous preclinical schedule, among other factors, are major hindrances to the effective integration of ultrasound into preclinical curriculums [4,10]. Therefore, the concept of peer tutoring offers an intriguing solution to address this educational gap, enabling students to actively engage in teaching and learning from their peers while providing a framework for continued preclinical ultrasound education through peer tutoring. 

This paper endeavors to investigate the efficacy of STs trained by other students in imparting the fundamentals of one of the most straightforward ultrasound uses: the musculoskeletal (MSK) ultrasound of the upper extremity. Other studies exploring the use of STs to educate on MSK ultrasound exist [15], in addition to other studies with emphasis on different body systems, including cardiac, vascular, and abdominal ultrasounds [16-18]. However, none explored the specific potential of a peer tutoring system that is completely independent of formal ultrasound instruction by faculty members and driven solely by student-taught tutors. Our study seeks to contribute valuable insights into optimizing limited ultrasound exposure and resources in the preclinical years by exploring the role that students can have in educating each other without the use of faculty resources. Ultimately, this research aims to advance medical education and promote a culture of peer-driven learning, ensuring that future physicians are equipped early on with essential ultrasound skills required to excel in their clinical practice.

## Materials and methods

Sixty first-year medical students signed up to participate in the study. The first session consisted of four student groups, split randomly between two faculty ultrasound instructors (UIs) and two STs. The secondary session also contained four student groups split randomly between two faculty UIs and two PTSTs. The students initially signed up through a Google Form (Google LLC, Mountain View, California, United States), and the roster was extracted to a Google Sheet (Google LLC, Mountain View, California, United States). The students were assigned a random number from 1 to 4, and then each of the numbers was randomly assigned to a teacher, which was also randomized in the same manner. The students were not assigned sequentially. All students who signed up (60 total) were randomized prior to the event starting. The randomization was performed in a blind manner to the identity of the teacher and students. Faculty instructors were from the California University of Science and Medicine (CUSM) in Colton, California, who had expert knowledge of ultrasound techniques and regularly used them in their field of practice. The STs consisted of third-year medical students who were educated on MSK ultrasound by the faculty UIs, while the PTSTs were educated solely by the third-year STs, with no previous exposure to UI instruction. Each of the student groups was randomly assigned to a different teaching group (either PT-ST, ST, or UI). Each of the four teaching groups was randomly assigned one standardized patient who would provide their right forearm for sonographic imaging. The Institutional Review Board (IRB) of the CUSM issued approval HS-2022-26.

Each ultrasound workshop session was split into two 30-minute segments. The first 30 minutes consisted of educators introducing participants to a standardized basic introduction to general ultrasound technique, including how to appropriately hold the ultrasound probe, how to appropriately apply ultrasound gel to the area of interest, and how to identify correct image orientation (educators were told to keep this segment brief and 10 minutes maximum if needed). Each station was set up with the same ultrasound equipment, including one Butterfly iQ3 ultrasound probe with a MacOS app on an iPad interface, which was previously randomized between each room. Each ultrasound probe and iPad were set to the MSK setting, which included the same depth and gain. The next 15 minutes of the initial 30-minute session included a visual example with commentaries given by the educator, with emphasis on the correct identification of structures within the volar surface of the forearm. During this time, participating first-year students were shown by their respective educators where to find and how to screenshot/label/identify the median nerve, radial nerve and artery, and ulnar nerve and artery accurately. Once the demonstration was complete, any remaining time for the initial 30-minute introduction was used as a time for participants to practice their ultrasound techniques on the standardized patients and students were given the opportunity to ask questions regarding accurate sonographic identification of volar structures. Once the initial 30-minute introduction of the session had ended, the students were asked to identify all five structures without assistance. The participants not actively being assessed were asked to step at least 5 feet away from the assessment table and were told not to speak, give feedback, or actively study the technique of actively participating students. Students were told to take screenshots and label their images as accurately as possible, identifying the aforementioned volar structures on a standardized patient without any feedback. Students were allowed to adjust depth and gain parameters during their assessment, but all settings were reset between student assessments to standardize each attempt. The labeled screenshot images of volar structures were graded based on the ability to clearly visualize the intended structures.

Immediately after being assessed, the participants were asked to individually complete a mandatory seven-question confidence survey that could be accessed via a QR code. This survey contained seven questions asking students to rate their confidence level in identifying anatomical structures and general ultrasound techniques after their workshop experience. Possible responses for the confidence questions were based on a Likert scale, with the options being "Not Confident" (1), "Slightly Confident" (2), "Somewhat Confident" (3), "Quite Confident" (4), and "Very Confident" (5). In addition, the students were given the option to answer a two-question pathology quiz in which each question showed a specific sonographic image of the volar forearm. The students were instructed to state "Yes" or "No" when asked if the given sonographic image was indicative of a pathology and if the student answered "Yes," to explain why they believed pathology to be present within the given image. The participants were awarded one point for an accurate answer and an additional point for an acceptable explanation of why they believed pathology to present. The first question showed an image of a median nerve neuroma and acceptable explanations were assessed by accuracy and relevance. Examples of correct answers included “Nerve appears hypoechoic” and “The median nerve appears compressed.” 

Before grading, the collected images were de-identified by labeling the image with the student's last four digits of their phone number, which could then be matched to the user retroactively. Images were then graded anonymously and independently by non-teachers (graders were not PTSTs or UIs) using a binary grading rubric: students were given one point if the structure was clearly visible and labeled correctly and zero points if it was the incorrect structure, if the structure was not present in the image, or the structure was labeled incorrectly. The rubric depends on whether the required structure (i.e., the median nerve) was present anywhere in the image submitted by the student. The graders used widely accepted characteristics and definitions used in ultrasound medical education (hyperechoic, “honeycomb” structure typical of nerves and circular structures with hypoechoic centers typical of arteries) to definitively grade the images. We observed that all grades were found to be unanimous between graders even with anonymous, independent grading at different time points. This is likely due to the binary nature of the established rubric and the simplicity of identifying whether the labeled structure was present within the screenshot. Therefore, given the unanimity observed between independent graders, the variance between graders was determined to be negligible with no further necessity to measure the statistical inter-rater reliability.

Chi-squared analysis with Yates correction was used in analyzing the differences in image accuracy between UIs and PTSTs and analyzing the pathology responses between UIs and PTSTs. We also used the Mann-Whitney U-test to compare the confidence questions between UIs and PTSTs.

## Results

**Figure 1:**
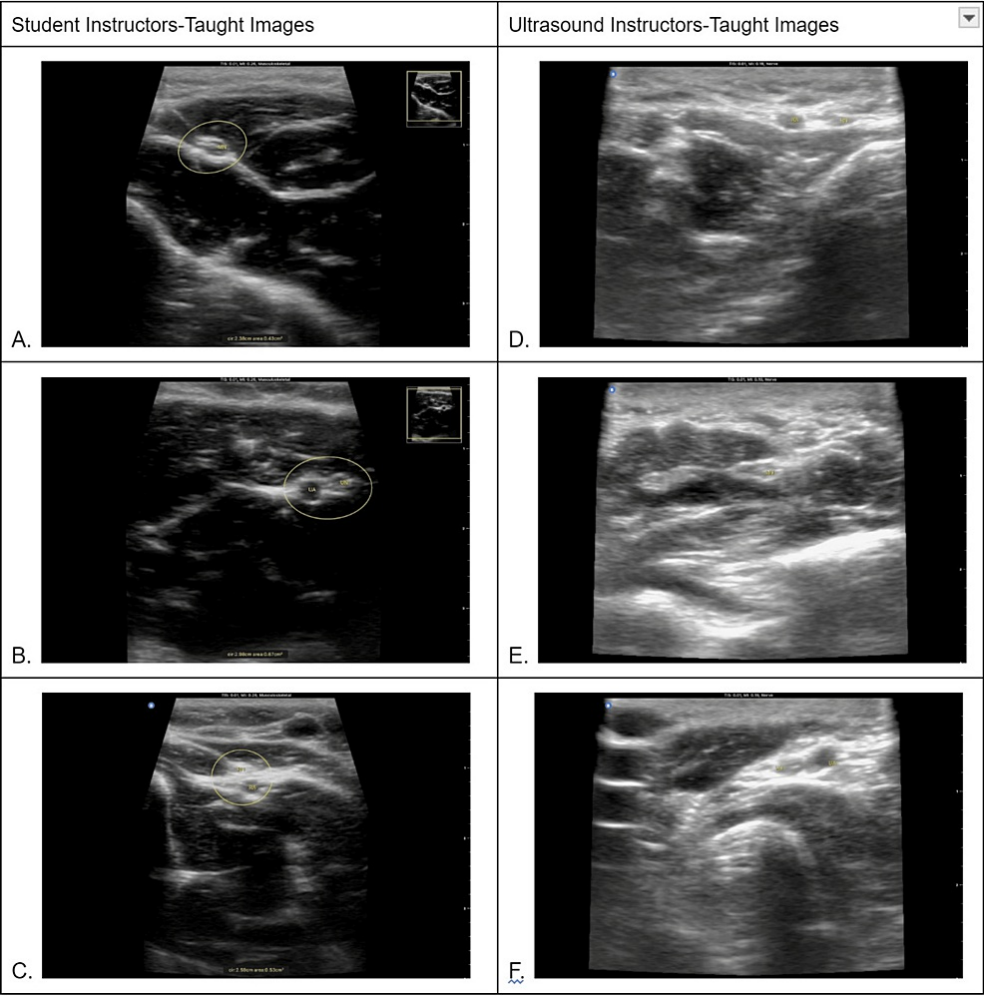
Side-by-side comparison of correctly labeled student ultrasound images between teaching groups. Images A, B, and C are images captured by students taught by STs and identify the median nerve, ulnar nerve, and artery, and radial nerve, respectively. Images D, E, and F were captured by students taught by UIs and identify the median nerve, ulnar nerve and artery, and radial nerve, respectively.

As mentioned previously, a total of 60 students were recruited via email with a Google Form link acting as a sign-in sheet prior to data collection so that rooms and teaching groups could be scheduled and randomized. At the time of data collection, 10 participants were absent despite being assigned as participants and being already randomized to a teaching group. Nine of the absent students had been assigned to the UI sessions, and one of the absent students had been assigned to one of the student tutor (PTST) sessions. However, to preserve the randomization of the participants, the groups were not adjusted in response to unexpected participant absences. As a result, there were 29 students who were taught by PTSTs and 21 students who were taught by UIs. Figure 1 shows the comparison between the average quality of ultrasound images taken by students taught by PTSTs and UIs, respectively. There was no significant difference in image quality when assessing the identified structures. 

As noted in Table 1 and Table 2, 93% of the students taught by PTSTs were able to identify the radial artery, while 95% of the students taught by UIs were able to identify the radial artery. For the median nerve, 93% of the students in the PTST group were able to identify compared to 76% of students in the UI group. Moreover, 97% of students in the PTST group were able to identify the ulnar artery, while 81% of students in the UI group were able to identify. The ulnar nerve showed only a 62% correct identification rate of students in the UI group compared to 79% for students in the PTST group. The structure that students found most difficult to identify was the ulnar nerve. Table 1 displays no statistically significant difference in identification accuracy between the UI and PTST student groups (p > 0.05). When using the Mann-Whitney U-rank test, there remained no statistically significant difference between the accuracy of the students taught by STs compared to students taught by UIs (Table 2). Figure 1 depicts a side-by-side comparison of student-captured images with labeled structures to provide samples of captured and labeled images from the corresponding teaching groups. The images labeled A, B, and C in Figure 1 are samples of images captured by students taught by PTSTs. The images labeled D, E, and F in Figure 1 are corresponding images from students taught by UIs. The students were instructed to place the appropriate two-letter label (“MN” for median nerve) superimposing the structure of interest. Figure 1 serves to provide a direct visual comparison between labeled images captured by students belonging to the two different teaching groups. This figure also serves to display samples of correctly labeled images taken by students from each teaching group. Students in both teaching groups were given the option of encircling the structure of interest; however, while independently grading the images according to the aforementioned rubric, points were awarded if the structure was clearly visible and labeled correctly regardless of whether the structure was circled in the image. Therefore, the circles found in Figure 1 can be disregarded. 

**Table 1:** Comparison of accuracy, using chi-squared with Yates correction of ultrasound identification when taught by STs or UIs. ST = student tutor; UI = ultrasound instructor

Structures	Teacher	Could identify (# of students)	Could not identify (# of students)	X^2 (df =1)	P-value
Radial artery	ST	27	2	0.098	0.7538
	UI	20	1		
Median nerve	ST	27	2	1.659	0.1977
	UI	16	5		
Ulnar artery	ST	28	1	1.788	0.1812
	UI	17	4		
Ulnar nerve	ST	23	6	1.069	0.3012
	UI	13	8		

**Table 2:** Comparison of accuracy using Mann-Whitney U-rank tests when taught by STs or UIs. ST = student tutor; UI = ultrasound instructor

Structures	Teacher	Could identify (# of students)	Could not identify (# of students)	P-value	U-value	Z-value
Radial artery	ST	27	2	0.7744	298	-0.2867
	UI	20	1			
Median nerve	ST	27	2	0.09538	356	1.6677
	UI	16	5			
Ulnar artery	ST	28	1	0.07547	352	1.7776
	UI	17	4			
Ulnar nerve	ST	23	6	0.1846	357.5	1.3267
	UI	13	8			

**Figure 2:**
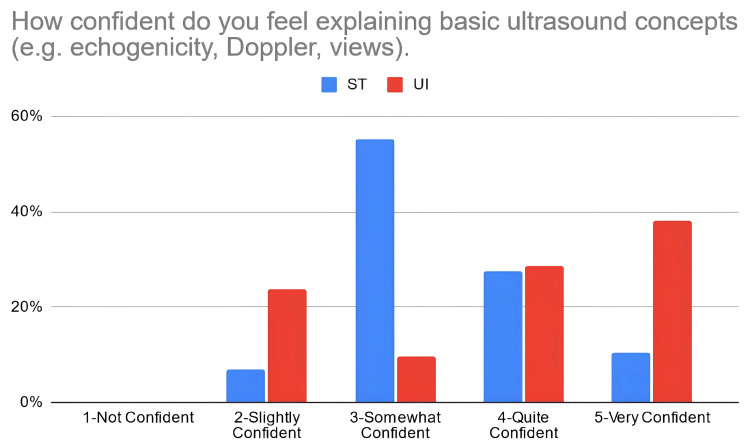
Self-reported confidence levels of students in explaining basic ultrasound concepts according to the teaching group ST: peer-taught student tutors, UI: ultrasound instructors

**Figure 3:**
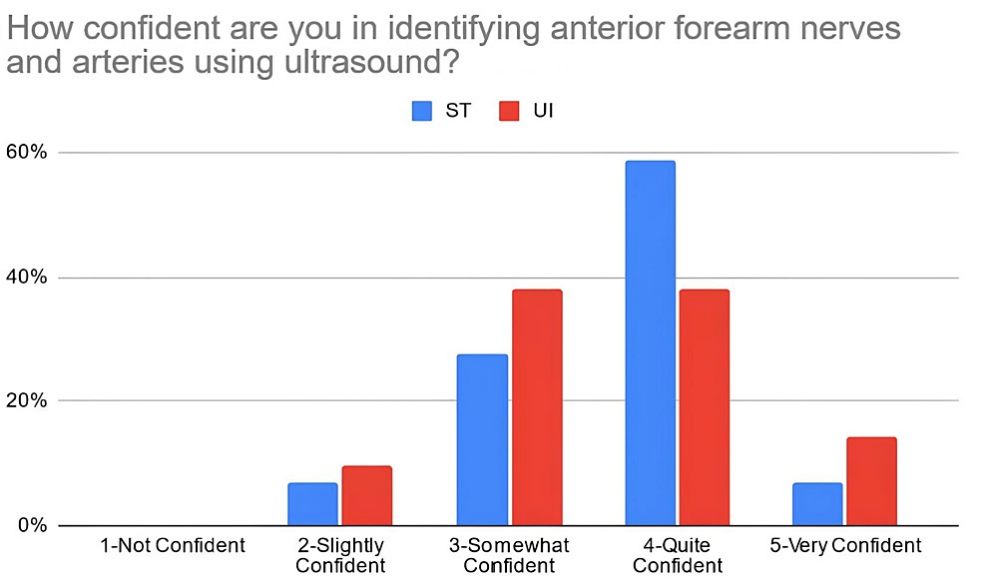
Self-reported confidence levels of students in identifying anterior forearm nerves and arteries according to the teaching group ST: peer-taught student tutors, UI: ultrasound instructors

**Figure 4:**
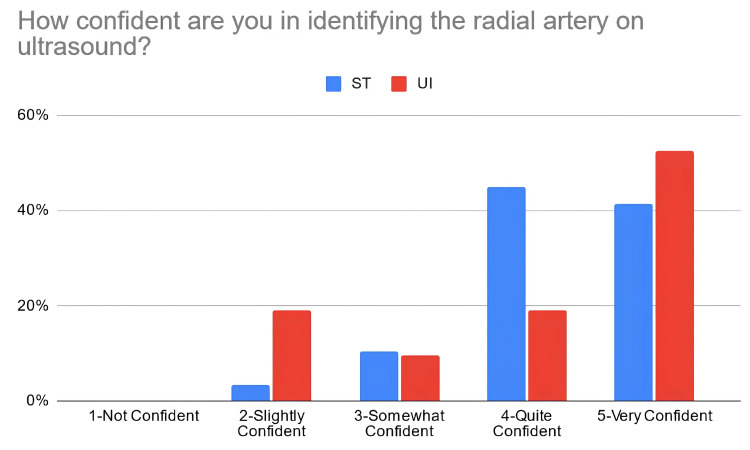
Self-reported confidence levels of students in identifying the radial artery according to the teaching group ST: peer-taught student tutors, UI: ultrasound instructors

**Figure 5:**
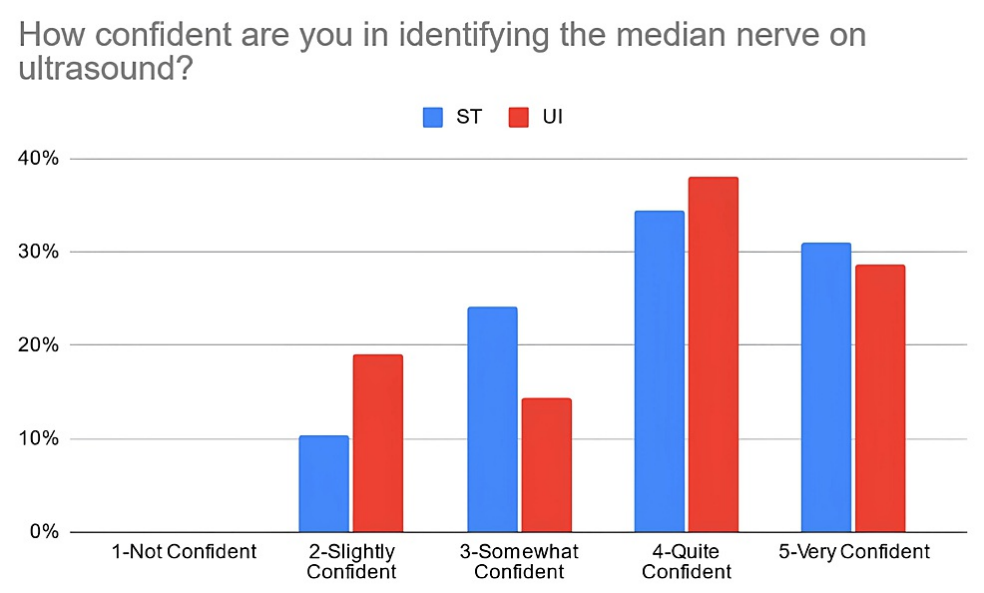
Self-reported confidence levels of students in identifying the median nerve according to the teaching group ST: peer-taught student tutors, UI: ultrasound instructors

**Figure 6:**
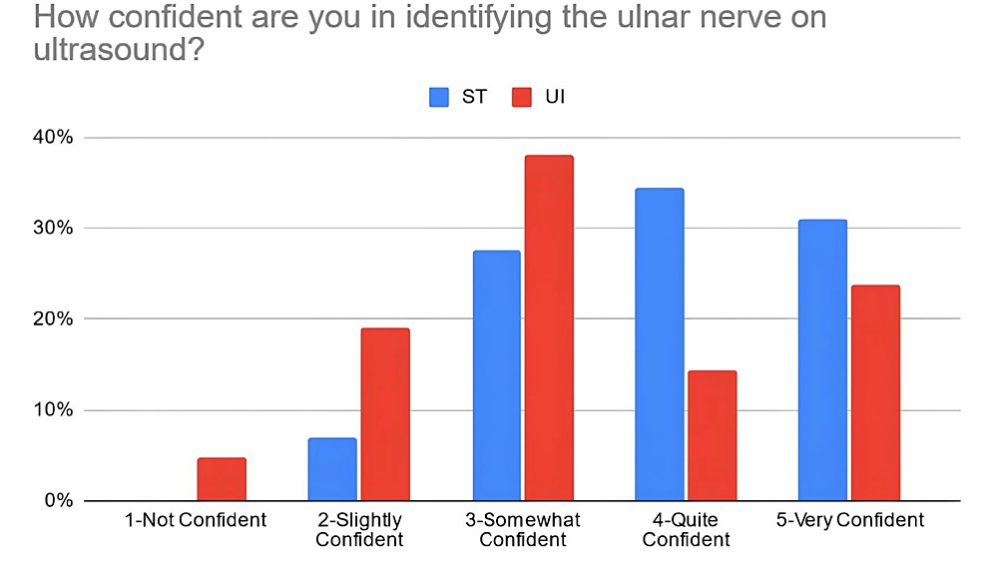
Self-reported confidence levels of students in identifying the ulnar nerve according to the teaching group ST: peer-taught student tutors, UI: ultrasound instructors

**Figure 7:**
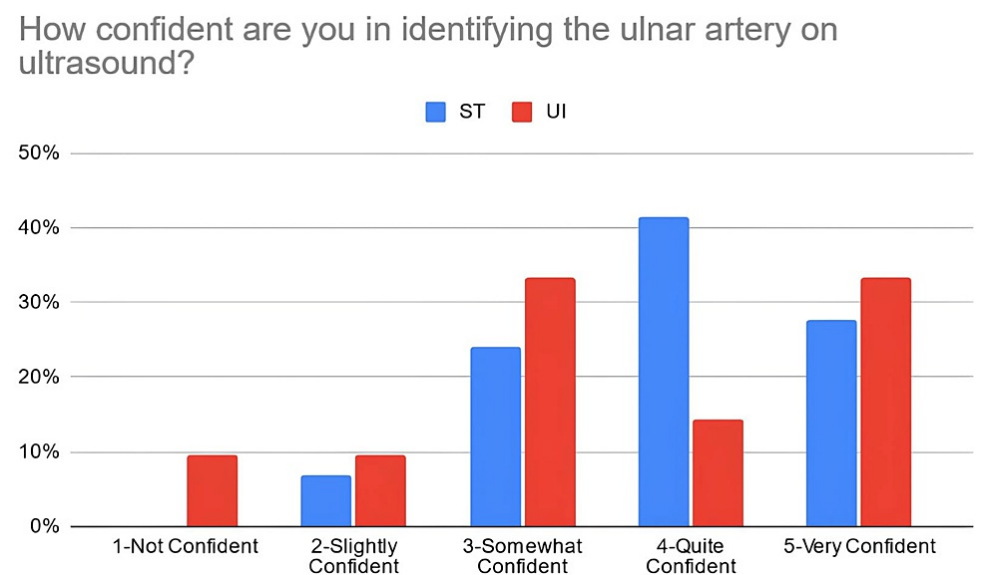
Self-reported confidence levels of students in identifying the ulnar artery according to the teaching group ST: peer-taught student tutors, UI: ultrasound instructors

**Figure 8:**
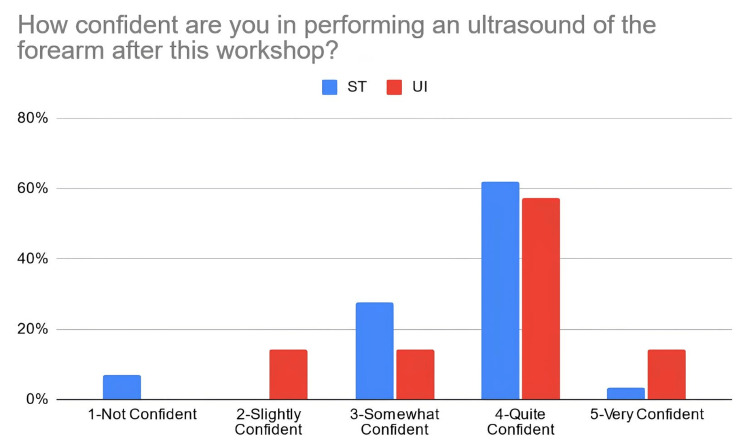
Self-reported confidence levels of students in performing an ultrasound of the forearm according to the teaching group ST: peer-taught student tutors, UI: ultrasound instructors

According to Table 3, 38% of the students in the PTST student group reported that they were confident in explaining basic ultrasound concepts after the workshop, compared to 67% of students in the UI student group. Moreover, 66% of PTST-taught students reported that they were confident in identifying volar nerves and arteries using ultrasound in comparison to 52% of UI-taught students. Eighty six percent (86%) of the ST-taught students reported that they were confident in identifying the radial artery on ultrasound compared to 71% of students in the UI student group. The percentage of students reporting confidence for identifying the median nerve was relatively similar for the PTST-taught students compared to UI-taught students, at 66% and 67%, respectively. For ulnar nerve/artery identification, 69% of the students in the PTST student group agreed that they were confident in identifying these structures. In comparison, 38% of the students belonging to the UI group reported confidence with identifying the ulnar nerve, and 48% were confident in identifying the ulnar artery. Sixty-six percent (66%) of the PTST-taught students reported that they felt more confident in the general use of the ultrasound modality after the workshop, compared to 71% of UI-taught students. Figures 2-8 display these findings in bar graph representations of student confidence according to instructor type, represented by percentage of student answers for each category.

**Table 3:** Student confidence in identifying volar arm structures and general ultrasound knowledge when taught by PTSTs and UIs. PTST: peer-taught student tutors, UI: ultrasound instructors

		Student confidence level responses (# of students)				
Question	Teacher	Not confident	Slightly confident	Somewhat confident	Quite confident	Very confident
How confident do you feel explaining basic ultrasound concepts (e.g., echogenicity, Doppler, views)?	PTST	0	2	16	8	3
	UI						0	5	2	6	8
	How confident are you in identifying anterior forearm nerves and arteries using ultrasound?	PTST	0	2	8	17						2
		UI					0	2	8	8	3	
How confident are you in identifying the radial artery on ultrasound?		PTST	0	1	3	13						12
		UI					0	4	2	4	11	
	How confident are you in identifying the median nerve on ultrasound?	PTST	0	3	7	10						9
		UI					0	4	3	8	6	
How confident are you in identifying the ulnar nerve on ultrasound?		PTST	0	2	8	10						9
		UI					1	4	8	3	5	
	How confident are you in identifying the ulnar artery on ultrasound?	PTST	0	2	7	12						8
		UI					2	2	7	3	7	
How confident are you in performing an ultrasound of the forearm after this workshop?		PTST	2	0	8	18						1
		UI					0	3	3	12	3	

Table 4 compares perceived confidence levels in students between the different teaching groups and demonstrates no statistically significant differences in how these two groups responded to these statements when using the Mann-Whitney U-rank test. 

**Table 4:** Comparison of confidence levels using Mann-Whitney U-rank tests when taught by STs or UIs. ST: peer-taught student tutors, UI: ultrasound instructors

Questions	Teacher	Median confidence level	Confidence level range	P-value	U-value	Z-value
How confident do you feel explaining basic ultrasound concepts (e.g. echogenicity, Doppler, views)?	ST	3	2-5	0.1397	232	-1.4768
	UI	4	2-5			
How confident are you in identifying anterior forearm nerves and arteries using ultrasound?	ST	4	2-5	0.6373	327	0.4715
	UI	4	2-5			
How confident are you in identifying the radial artery on ultrasound?	ST	4	2-5	0.9662	307	0.04235
	UI	5	2-5			
How confident are you in identifying the median nerve on ultrasound?	ST	4	2-5	0.8133	316.5	0.2362
	UI	4	2-5			
How confident are you in identifying the ulnar nerve on ultrasound?	ST	4	2-5	0.0844	389.5	1.7257
	UI	3	1-5			
How confident are you in identifying the ulnar artery on ultrasound?	ST	4	2-5	0.3737	348.5	0.8896
	UI	3	1-5			
How confident are you in performing an ultrasound of the forearm after this workshop?	ST	4	1-5	0.4674	271.5	-0.7267
	UI	4	2-5			

Table 5 demonstrates the comparison of results between the teaching groups of students who participated in the optional pathology-based ultrasound questions. Approximately 15 ST-taught students and 11 UI-taught students answered the two pathology questions. Eighty percent (80%) of the ST-taught students answered the first pathology question correctly, compared to 64% for the UI-taught students. For the radial artery-related pathology question, 67% of the students taught by STs answered it correctly, and 64% of the students taught by UIs answered it correctly. Using chi-square with Yates correction, there is no sufficient evidence to justify an association between the ability to answer pathology-based ultrasound questions and instructor type.

**Table 5:** Comparison of student accuracy between teaching groups in answering post-workshop novel sonographic pathology questions using chi-squared with Yates correction ST: peer-taught student tutors, UI: ultrasound instructors

Question	Teacher	Answered correctly (# of students)	Answered incorrectly (# of students)	X^2 (df =1)	P-value
Median nerve pathology question	ST	12	3	0.232	0.6299
	UI	7	4		
Radial artery pathology question	ST	10	5	0.026	0.8725
	UI	7	4		

## Discussion

The goal of this study is to compare the capabilities of STs and UIs as teachers of the basics of MSK ultrasound. The easiest way to demonstrate whether one teaching group outperformed the other is through the correct identification of sonographic structures. This comparison is presented in Tables 1 and 2, which demonstrate no statistically significant difference in accuracy between the two student groups. There remained no statistically significant difference in accurate identification across all four tested structures. The lack of statistically significant differences between the two student groups suggests several possibilities, one of which is that first-year medical students who participated in this study were equally capable of learning to identify basic volar forearm ultrasound structures when led by either UIs or STs. The efficacy of student-led UIs has been previously explored and has also shown similar positive outcomes, such as improved skill confidence and improved assessment scores, when compared to other types of UIs [16,19,20]. Our findings show that the ability to identify the structures of the forearm had no correlation to what type of teacher was available, presenting the possibility that PTSTs may be used in lieu of UIs with minimal variation in the resultant sonographic capabilities of novice students.

Similarly, as shown in Table 5, there was no justifiable association between the ability to answer pathology-related questions and the teacher type the students were assigned. This segment of the workshop was designed to reveal any differences in the concept of “transfer” or the ability to apply knowledge, skills, and abilities to novel contexts and tasks that have not been previously experienced [20]. The lack of a significant difference in the ability to answer novel ultrasound-related pathology questions further reinforces the similarity in educational value between UIs and STs. 

While a majority of ST-taught students stated that they were “somewhat confident” of their basic ultrasound concepts, there were significantly more UI-taught students who thought themselves to be “very confident” in explaining basic ultrasound concepts. This finding suggests that faculty UIs may have an advantage over STs in instilling confidence in students’ perceived sonographic ability. This could be due to a number of factors, one of which could be that students perceive UI as seasoned experts in their field, which in turn bolsters the students’ confidence in sonographic ability since they perceive to be learning from experts rather than their peers. However, other studies have shown that STs instill more confidence in medical students in the setting of ultrasound instruction [18-21,22,23]. One of these studies stated that their data and medical student evaluations suggested that this may be due to peer tutors’ ability to conform their teachings to fit the learning style of other students [24]. We emphasize that there remained no statistically significant difference in the accuracy of the imaging when comparing students taught by STs or UIs. This finding can of course be variable between different educators, since each UI or ST may have their own individual strengths that affect students’ levels of perceived confidence. 

Our study displays the efficacy of using STs to provide basic and introductory ultrasound experience to novice medical students. It goes without saying that UIs have a wealth of experience that STs lack, but at the level of basic sonographic identification, STs show promising effectiveness as educators of novice users when compared to faculty UIs. This near-peer teaching strategy has shown successful implementation in other point-of-care ultrasonography (POCUS) curricula, including improved student knowledge, ultrasound skills, and clinical transferability of learned topics [20,25]. Given these findings, the use of STs to provide early exposure to novice ultrasound students in their preclinical years of medical school would allow for a dramatic increase in resources available to bolster both the sonographic ability and perceived confidence in medical students. A previous study has also demonstrated the importance of early exposure to ultrasound, showing a marked increase in confidence and knowledge in performing a focused assessment with sonography in trauma (FAST) exam in medical students who had previous ultrasound training [26]. Not only would coordinated peer tutoring ultrasound workshops, similar to this experiment, increase overall levels of sonographic competency, but it would also facilitate the establishment of a more collaborative medical school community through a small group format that may be lacking in other areas of preclinical curriculum [11,27]. A proposed peer tutoring framework could include a UI-led session to educate STs, and as demonstrated in this experiment, these STs could further educate other future tutors, creating a multiplicative increase in the amount of experienced ultrasound PTs. Several peer tutoring models within the context of medical school curricula have been described with simple steps of integration [2,5,28], providing an easily reproducible peer tutoring framework. Within these frameworks, PTSTs will allow for an earlier facilitated exposure to the basic concepts of ultrasound, allowing for a coordinated increase in ultrasound competency and competence. 

One limitation of the study was the inclusion criteria for the population. The students consisted solely of first-year medical students who had recently started the MSK learning block of their curriculum. Each participating student had self-volunteered for the workshop, which was advertised by members of the ultrasound interest group at CUSM. The self-selection of participants could be a potential limitation of the study, as these students choosing to participate might have resulted in a sample that is not representative of the entire first-year cohort. The generalizability could be limited, as participants may have been more likely to be high-achieving or particularly interested in ultrasound, in comparison to their peers, since they chose to be participants of this study. Given the specific inclusion criteria of this study, the generalizability of these results may be limited. Furthermore, there was only a limited number of UIs and STs available for each session, which restricted the number of total students who were able to participate in the study. Students were, however, assigned randomly to their teacher type. Nonetheless, some students did not report to their designated times for the study, which could have skewed data as UIs and STs did not have the same number of total students taught. The small sample size of this study also contributes to the limited generalizability of the results. A consideration for possible bias in this study would be the Hawthorne effect (variability in results due to a subject’s awareness of being observed), since STs and UIs remained in the room when the students were taking their unaided screenshots of the volar structures. Some students did not collect recordings for the radial nerve, while others did, leading to the disqualification and exclusion of the radial nerve from the study. In addition, the binary response to the pathology questions (yes or no) could be considered a "forced choice," which is a limitation of our study. If a third choice, such as "Not Sure," were added, the responses to the survey may have been different and led to a different interpretation considering the educational aims of this study. Lastly, the participants performed their ultrasound studies on four different standardized patients, in which small anatomic variabilities could have caused variability in the results of the study. This possibility was minimized by sonographically reviewing all standardized patients' volar structures prior to the sessions with the students; no major anatomic variabilities were identified between standardized patients. 

For future studies that seek to reproduce these results, increasing the total number of participants may strengthen the power of the study. Future studies could also enhance the generalizability of the results by comparing the academic performance of participants with that of the general first-year cohort to determine if self-selected participants are indeed higher performing. In addition, repeating the study with a consistent cohort who is followed for multiple ultrasound sessions with either an ST or a UI would provide longitudinal data to help support the conclusions found in this paper.

## Conclusions

Ultrasound competency is becoming an increasingly critical skill for medical students to learn given its versatility as a diagnostic tool in a variety of medical specialties. Our study supports the interpretation that the capability of first-year medical students to learn novice MSK sonographic identification is independent of whether the educator is a PTST or UI. This interpretation reveals a promising avenue toward the integration of the fundamentals of ultrasound identification early in medical education with little to no concern for the exhaustion of institutional resources. An integrated tutoring system led solely by PTSTs would allow for a much larger number of students to gain hands-on experience with ultrasound technology in their preclinical years. This early exposure to ultrasound techniques can greatly benefit students who are interested in medical specialties that frequently make use of the diagnostic capabilities of POCUS. Along with the other well-documented benefits of the utilization of student tutors in medical school, a peer tutoring system centered on ultrasound skills designed in the way this study describes can be an effective, resource-sparing integration of ultrasound into preclinical curricula. Implementation of such a system may provide medical students with a more robust foundation in one of the most widely used diagnostic tools in medicine, which in turn could be a difference in positively affecting patient outcomes. Further studies that have a more longitudinal cohort and studies with larger sample sizes are needed to better demonstrate the efficacy of PTSTs in medical education and specifically ultrasound basics.
